# Variables associated with sleep quality in chronic tension-type headache: A cross-sectional and longitudinal design

**DOI:** 10.1371/journal.pone.0197381

**Published:** 2018-05-17

**Authors:** Elena Benito-González, Maria Palacios-Ceña, Juan J. Fernández-Muñoz, Matteo Castaldo, Kelun Wang, Antonella Catena, Lars Arendt-Nielsen, César Fernández-de-las-Peñas

**Affiliations:** 1 Department Physical Therapy, Occupational Therapy, Rehabilitation and Physical Medicine, University Rey Juan Carlos, Alcorcón, Spain; 2 Center for Sensory-Motor Interaction (SMI), Department of Health Science and Technology, School of Medicine, Aalborg University, Aalborg, Denmark; 3 Department of Psychology, Universidad Rey Juan Carlos, Alcorcón, Spain; 4 Master in Sport Physiotherapy, University of Siena, Siena, Italy; 5 Poliambulatorio Fisiocenter, Collecchio, Parma, Italy; Taipei Veterans General Hospital, TAIWAN

## Abstract

**Objective:**

To investigate variables associated at baseline (cross-sectional design) and at one year (longitudinal design) with the quality of sleep in chronic tension-type headache (CTTH).

**Methods:**

One hundred and eighty (n = 180) and 135 individuals with CTTH participated in the cross-sectional and longitudinal design respectively. Clinical features were collected with a 4-weeks headache diary at baseline and one-year follow-up. Sleep quality was assessed at baseline and 1-year follow-up with the Pittsburgh Sleep Quality Index. Anxiety and depression (Hospital Anxiety and Depression Scale-HADS), burden of headache (Headache Disability Inventory-HDI), quality of life (SF-36 questionnaire), and pressure pain thresholds (PPTs) at trigeminal, extra-trigeminal and widespread area were assessed at baseline. Hierarchical regression analyses were conducted to determine the associations between variables at baseline and 1-year follow-up with sleep quality.

**Results:**

At baseline positive correlations between sleep quality and headache intensity, headache frequency, headache duration, emotional and physical burden of headache and depression were observed. The regression analyses found that depression and emotional burden of headache explained 27.5% of the variance in sleep quality at baseline (r^2^ = .262; F = 23.72 P < .001). At one-year, sleep quality was significantly associated with baseline burden of headache, depression, widespread PPTs, vitality and mental health domains. Regression analyses revealed that vitality, PPT over the second metacarpal and PPT over the neck explained 30.0% of the variance of sleep quality at one-year (r^2^ = .269, F = 9.71, P < .001).

**Conclusions:**

It seems that sleep quality exhibits a complex interaction in individuals with CTTH since depression and the emotional burden were associated with sleep quality at baseline, but vitality and PPTs over extra-trigeminal areas were associated with the quality of sleep at one-year.

## Introduction

Tension-type headache (TTH) is a frequent pain disorder with a global prevalence of 42% in the general population [1] and showing an important socio-economic impact [2]. In the Global Burden of Disease Study, TTH was found to be the second most prevalent disorder in the world [3]. The general costs for TTH and migraine in Europe in 2010 were €13.8 billion [4] and were mostly associated to the chronic forms.

The most accepted model for TTH pathology proposes abnormality in nociceptive pain processing by including peripheral and central sensitization mechanism [5]. In fact, it is suggested that the pain in the episodic form has a peripheral component whereas central nervous system factors play a more relevant role in the chronic form of tension type headache (CTTH) [5]. The presence of central sensitization is generally attributed to long-lasting prolonged bombardment of nociceptive afferences or stimulus arriving to the central nervous system and to the trigemino-cervical nucleus caudalis. This process is particularly important in CCTH patients, since a higher frequency of headache attacks is able of triggering hyperalgesic responses in the central nervous system and promoting pain [5]. Additionally, it is important to consider that not only headache attacks, but also several physical, physiological or emotional factors may also influence the excitability of the central nervous system observed in subjects with CTTH and could contribute to the development and/or maintenance of the symptoms. Among these potential factors, depression, anxiety, or sleep disorders play a relevant role in the process of sensitization of central nervous pathways since they are able of triggering hyperalgesic responses by increasing pressure pain hypersensitivity [6]. In fact, current research suggests that one of the main manifestations of sensitization mechanisms in patients with CTTH is the presence of widespread pressure pain hyperalgesia, which is not particularly present in those with the episodic form [5].

It seems that stress and sleep disturbances are the most common trigger factors for headache attacks in individuals with headache [7] and, when combined, they exhibit an additive effect [8]. In addition, individuals with headache often exhibit co-morbid anxiety and depression [9] and sleep disturbances [10] and that these disorders are more present in those with chronic headaches [11,12]. In fact, the presence of depression and anxiety has been related to worse quality of life in subjects with chronic headaches [13]. Additionally, anxiety and depression are well known factors also associated with sleep disturbances [14] and it has been recently suggested that combined load of emotional stress and sleep-related symptoms can be an etiological factor for headache [15].

Since all these factors could interact by increasing stimulating the central nervous system in patients with CTTH and; hence, contributing to the maintenance of central sensitization; a better understanding of the potential associations between depression, anxiety, headache, related-disability, and sleep quality in subjects with CTTH can assist clinicians in determining better therapeutic programs. Since central sensitization, mood disorders, including anxiety and depression, and sleep disturbances are more associated to the frequency of headache attacks [11,12], we focused the current study just in the chronic form of the disease. No study has previously investigated variables explaining sleep quality in individuals with CTTH in a longitudinal design. Therefore, the purposes of the current study were to investigate potential variables associated at baseline (cross-sectional design) and at one year (longitudinal design) with sleep quality in a cohort of patients with CTTH.

## Material and methods

### Study design

The current analysis is included as part of a multicenter international headache study. Some patients from the current study were also included in a previous part of the study which data have been already published [16, 17]. This study presents new data by including more patients, different outcomes and statistical analysis, and a longitudinal design.

### Participants

Consecutive subjects with TTH were recruited from different university-based hospitals between January 2015 and June 2016. Diagnosis was conducted according to the International Classification of Headache Disorders, third edition (ICHD3 beta, 2013) down to third-digit level (codes 2.2, 2.3) by neurologist’s expert in headaches [18]. To be included, participants had to describe typical pain features of TTH: bilateral location, pressing or tightening pain, moderate intensity (≤6.5 on a 10 points numerical pain rate scale, NPRS) and no aggravation of pain during physical activity. Further, participants should report neither more than one symptom, including photophobia, phonophobia or mild nausea as requested by the ICHD-III criteria [18].

Participants were excluded if presented: 1, episodic headaches; 2, other primary or secondary headache including medication overuse headache [18]; 3, history of head or neck trauma (i.e., whiplash); 4, cervical herniated disk or cervical osteoarthritis; 5, any systemic degenerative disease, e.g., rheumatoid arthritis, lupus erythematous; 6, diagnosis of fibromyalgia; 7, had received anesthetic blocks the previous 6 months; 8, physical treatment in the neck/head received the previous 6 months; or, 9, pregnancy.

All participants read and signed a consent form prior to their participation. The local Ethics Research Committee of each country approved the study (Universidad Rey Juan Carlos URJC23/2014, Hospital Universitario Fundación Alcorcon HUFA14/104, Aalborg University N20140063, Universita Degli Studi Di Urbino Carlo CESU5/2015).

### Sleep quality

The main outcome, sleep quality was assessed with the Pittsburgh Sleep Quality Index (PSQI), one of the questionnaires most commonly used [19]. This questionnaire evaluates the quality of sleep over a 1-month period by including 19 self-rated questions and other 5 questions answered by bed/room mates. Item use varying response categories recording usual bed time, usual wake time, number of actual hours slept and number of minutes to fall asleep. All questions are answered on a Likert-type scale (0–3). The sum of all answers is transformed into a global score (0–21) where higher score indicates worse sleep quality [20]. A total score greater than 8.0 points is indicative of poor sleep quality [19]. The PSQI has shown good internal consistency and test-retest reliability [21]. In the current study, sleep quality was assessed at baseline and at 1-year follow-up.

### Headache outcomes

Key elements of the clinical history, including headache-family history, headache features, temporal pattern, and medication were assessed. A headache diary for 4 weeks was used to substantiate the diagnosis and to record the headache clinical features [22,23]. On this diary, patients registered the number of days with headache (days per week), the intensity of the headache attack on an 11-points numerical pain rate scale [24] (NPRS; 0: no pain, 10: maximum pain), and the duration of each headache attack (hours per day). The headache diary was assessed at baseline and at 1-year follow-up.

### Headache burden

The Headache Disability Inventory (HDI) assesses the burden of headache using 25 items that inquire about the perceived impact of headache on emotional functioning and daily life activities [25]. Possible answers for each item are *yes* (4 points), *sometimes* (2 point) or *no* (0 points). Thirteen items asses the emotional burden (HDI-E, maximum score: 52 points) and 12 items assess the physical burden (HDI-P, maximum score: 48 points) of headache. A greater score suggests greater burden of headache. The HDI has exhibited good stability at short and long-term [26]. The HDI was assessed only at baseline.

### Anxiety and depression

The Hospital Anxiety and Depression Scale (HADS) is a 14 items self-reported scale, 7 items for anxiety (HADS-A) and 7 items for depression (HADS-D), suggesting the presence of anxiety and depressive symptoms [27]. Each item scores on a Likert scale (0–3) giving a maximum of 21 points for each subscale [28]. The HADS has shown to have good validity and reliability in the general population [29] and in patients with headache [30]. The HADS was also assessed only at baseline.

### Health-related quality of life

Health-related quality of life was assessed at baseline with the Medical Outcomes Study Short Form 36 (SF-36) questionnaire. This questionnaire includes the following 8 domains (physical functioning, physical role, bodily pain, general health, vitality, social function, role-emotional, and mental health) where 0 represents the lowest quality of life and 100 the highest quality of life [31]. After summing the Likert-scaled items of each domain, it is scored from 0 (lowest health-related quality of life) to 100 (highest health-related quality of life) [32]. The Sf-36 was assessed only at baseline.

### Pressure pain thresholds

Pressure pain thresholds (PPTs), i.e., the pressure where a sensation of pressure changes to pain, were bilaterally assessed with a electronic pressure algometer (Somedic AB®, Farsta) over a trigeminal point (temporalis muscle), an extra-trigeminal point (C5 / C6 zygapophyseal joint) and 2 distant pain-free points (second metacarpal and tibialis anterior) to determine widespread pressure pain sensitivity [33]. PPTs were calculated only at baseline.

### Sample size calculation

The sample size was calculated using Ene 3.0® software (Autonomic University of Barcelona, Spain). The sample size calculation was based on detecting significant moderate correlations (r = 0.3) between the studied variables with an alpha level (α) of 0.05, and a desired power (β) of 95%. This generated a sample size of at least 130 participants.

### Statistical analysis

Means with their confidence intervals were calculated to describe the sample. The Kolmogorov-Smirnov test showed that all data get a normal distribution. Several Pearson product-moment correlation coefficients were first calculated to determine the relationship between the quality of sleep (dependent) and the remaining (independent) variables included in the study at baseline and at 1-year follow-up. This correlational statistical analysis was used to check for multicollinearity and shared variance between the outcomes.

Second, in those variables showing significant correlation with sleep quality, two separately hierarchical regression analyses were done to determine those variables that significantly contributed to the variance in the quality of sleep at baseline and at 1-year follow-up, separately. To examine the proportions of explained variance of sleep quality, 2 hierarchical regression analyses were conducted. Changes in *R*^2^ were reported after each step of the regression model to determine the association of each additional variable. Last, variables that significantly contributed to the quality of sleep at baseline or 1-year follow-up were selected for the inclusion into parsimonious final regression model. The significance criterion of the critical F value for entry into the regression equation was set at P<0.05.

## Results

A total of 200 individuals with headache were screened for possible eligibility criteria. Finally, 180 individuals with CTTH satisfied all eligibility criteria, agreed to participate and signed the informed consent at baseline. Twenty patients were excluded for the following reasons: co-morbid migraine (n = 13), episodic tension-type headache (n = 2) previous whiplash (n = 2), fibromyalgia (n = 2) and medication overuse headache (n = 1). One hundred and thirty-five (n = 135, 75%) of the included at baseline were also assessed at 12-months follow-up and therefore included within the main analysis (73% women, age: 45±14 years). **Table 1**summarizes demographic data of the sample at the beginning of the study.

**Table 1:** Demographic and clinical data of the sample at baseline.

	Mean	95%CI
**Clinical Data**		
Gender (F/M)	126 (70%) / 50 (30%)	
Age (years)	48	45–51
Years with headache	8.6	6.4–10.8
Headache intensity (NPRS, 0–10)	6.4	6.0–6.8
Headache frequency (days/month)	17.7	16.1–19.2
Headache duration (hours/attack)	7.4	6.6–8.2
**Psychological Data**		
HDI-E (0–52)	19.8	17.4–22.2
HDI-P (0–48)	23.4	21.2–25.6
HADS-D (0–21)	8.7	8.0–9.5
HADS-A (0–21)	11.6	10.8–12.4
Pittsburg Questionnaire (0–21)	8.4	7.6–9.2
**Health-related Quality of Life (SF-36 questionnaire, 0–100)**		
Physical Functioning	72.6	66.9–78.3
Physical Role	45.1	36.7–53.5
Bodily Pain	47.4	42.3–52.5
General Health	49.2	44.7–53.7
Vitality	46.5	41.5–51.5
Social Functional	63.2	57.7–68.7
Role Emotional	59.3	50.8–67.8
Mental Health	51.0	46.5–55.5
**Pressure Pain Thresholds (PPT, kPa)**		
PPT temporalis muscle	188	173–203
PPT C5-C6 joint	186	171–201
PPT second metacarpal	264	245–283
PPT tibialis anterior	402	369–435

HDI: Headache Disability Inventory (E: Emotional; P: Physical); HADS: Hospital Anxiety and Depression Scale (D: Depression)

### Baseline data: Cross-Sectional design

**Table 2**shows Pearson’s correlation coefficients of those variables significantly associated with sleep quality at baseline. Significant positive correlations between sleep quality (PSQI score) and headache intensity (r = .194; P = .02), headache frequency (r = .241; P < .001), headache duration (r = .165; P = .04), HDI-E (r = .374; P < .001), HDI-P (r = .259; P = .002) and HADS-D (r = .502; P < .001) were observed: the higher the headache intensity, the greater the headache frequency, the longer the headache duration, the higher the emotional and/or physical burden of the headache and the higher the depression level, the worse the sleep quality (**Table 2**).

**Table 2:** Pearson-Product moment correlation matrix for clinical and psychological variables statistically associated at baseline (n = 180).

	1	2	3	4	5	6
1. Pittsburg Questionnaire (0–21)						
2. Headache intensity (0–10)	.194*					
3. Headache frequency (days/month)	.241**	.146*				
4. Headache duration (hours/attack)	.165*	n.s	.249**			
5. HDI-E (0–52)	.374**	.222**	.387**	.350**		
6. HDI-P (0–48)	.259**	.172*	.266**	.237**	.825**	
7. HADS-D (0–21)	.502**	.239**	.313**	.192**	.535**	.404**

95%CI: 95% confidence interval

HDI: Headache Disability Inventory (E: Emotional; P: Physical); HADS: Hospital Anxiety and Depression Scale (D: Depression)

* P<0.05

** P<0.01

**Table 3**summarizes the hierarchical regression analysis conducted at baseline. In the analysis, depressive symptoms approximately contributed 24.5% (P < .001) whereas the emotional burden of the headache (HDI-E) contributed an additional 3% (P < .001) of the variance of sleep quality (PSQI). When combined, both variables explained 27.5% of the variance in sleep quality at baseline (r^2^ adjusted: .262, F = 23.72, P < .001)

**Table 3:** Summary of stepwise regression analyses to determine predictors of sleep quality at baseline (r^2^ = 27.5%).

Independent Variable	*B*	SE B	Β	t	F	P
Step 1						
HADS-D	.528	.082	.495	6.416	41.16	<0.001
Step 2						
HADS-D	.427	.093	.401	4.617	23.72	<0.001
HDI-E	.066	.030	.194	2.233		

Note: R^2^ = .245 for step 1 (adjusted R^2^ = .239); R^2^ = .275 for step 2 (adjusted R^2^ = .262)

HDI-E: Headache Disability Inventory (Emotional); HADS-D: Hospital Anxiety and Depression Scale (Depression)

### One-year follow-up data: Longitudinal design

**Table 4**summarizes Pearson’s correlation coefficients of the variables significantly associated with sleep quality (PSQI score) at 1-year follow-up. Significant positive correlations were observed between the quality of sleep at one-year and HDI-E (r = .282; P = .03), HDI-P (r = .226; P = .04), and HADS-D (r = .367; P < .001) at baseline: the higher the emotional and physical burden of the headache and the higher the depressive symptoms at baseline, the worse the sleep quality 1 year after (**Table 4**). Additionally, sleep quality at one-year was negatively associated with PPTs at all points at baseline (temporalis: r = -.285, P = .025; C5/C6: r = -.242, P < .001; second metacarpal: r = -.379, P < .001; tibialis anterior: r = -.293, P = .04), vitality (r = -.386; P < .001) and mental health (r = -.365; P < .001): the lower the widespread pressure hypersensitivity, the lower vitality and the lower mental health status at baseline, the worse the sleep quality one-year after (**Table 4**).

**Table 4:** Pearson-Product moment correlation matrix for functional and psychological variables at baseline statistically associated with sleep quality at one year (n = 135).

	1	2	3	4	5	6	7	8	9
1. Pittsburg Questionnaire (0–21)									
2. PPT temporalis muscle (kPa)	-.285*								
3. PPT C5-C6 joint (kPa)	-.242**	.711**							
4. PPT second metacarpal (kPa)	-.379**	.688**	.693**						
5. PPT tibialis anterior (kPa)	-.293*	.608**	.705**	.725**					
6. HDI-E (0–52)	.282*	-.260**	-241*	n.s	n.s				
7. HDI-P (0–48)	.226*	-.196*	-.206*	n.s	n.s	.780**			
8. HADS-D (0–21)	.367**	-.325**	-.364**	-.361**	-316**	.471**	.365**		
9. Vitality (SF-36, 0–100)	-.386**	.411*	.421**	.350**	.306**	-.474**	-.451**	-.615**	
10. Mental Health (SF-36, 0–100)	-.365**	.284**	.218*	.209*	n.s	-.359**	-.279**	-.730**	.476**

95%CI: 95% confidence interval

HDI: Headache Disability Inventory (E: Emotional; P: Physical); HADS: Hospital Anxiety and Depression Scale (D: Depression)

* P<0.05

** P<0.01

**Table 5**summarizes the hierarchical regression analysis conducted at one-year follow-up. In this analysis, baseline vitality approximately contributed 16.1%, PPT over the second metacarpal contributed an additional 8.6%, and PPT over the cervical spine contributed to the additional 5.3% (all, P < .001) of the variance on sleep quality (PSQI score) one-year after. When combined, these baseline outcomes explained 30.0% of the variance of sleep quality at one year (r^2^ adjusted: .269, F = 9.71, P < .001).

**Table 5:** Summary of stepwise regression analyses to determine predictors of sleep quality at 12 Months (r^2^ = 30.0%).

Independent Variable	*B*	SE B	B	t	F	P
Step 1						
Vitality	-.073	.020	-.402	-3.670	13.47	<0.001
Step 2						
Vitality	-.060	.019	-.329	-3.057	11.33	<0.001
PPT second metacarpal	-.013	.005	-.302	-2.806		
Step 3						
Vitality	-.071	.020	-.392	-3.625	9.71	<0.001
PPT second metacarpal	-.023	.006	-.515	-3.662		
PPT C5-C6 joint	-.019	.009	-.329	-2.265		

Note: R^2^ = .161 for step 1 (adjusted R^2^ = .149); R^2^ = .247 for step 2 (adjusted R^2^ = .225); R^2^ = .300 for step 3 (adjusted R^2^ = .269)

PPT: Pressure Pain Thresholds

## Discussion

The present study suggests that sleep quality exhibits a complex interaction in subjects with CTTH since we found that depressive symptoms and emotional burden of headache were significantly associated with sleep quality at baseline, but vitality and PPT over extra-trigeminal areas were longitudinally associated with the quality of sleep at one year.

A recent systematic review found moderate evidence indicating that depression, anxiety, poor sleep, medication overuse, stress and poor self-efficacy for managing pain were potential prognostic factor associated to poor prognosis and unfavorable outcomes from preventive drug treatment in chronic headaches [34]. The literature has described that headache frequency, anxiety, and depression are associated to sleep disturbances [14,35]. In our study, the frequency, the duration and the intensity of the headache attacks showed significant associations with sleep quality at baseline supporting the assumption that all headache features may be relevant for sleep quality; however, the regression analysis did not confirm such association. On the contrary, our results confirmed an association between the quality of sleep and depression in our sample of patients with CTTH, which agree with previous data in subjects with chronic pain [36] or with headaches [12]. Although a cross-sectional design does not allow determining the mechanisms involved in the relationship between depression and sleep quality, it has been suggested that headaches, sleep disturbances and depression share common brain mechanisms, i.e., hypothalamus, serotonin or melatonin neural circuits dysregulation [37]. Nevertheless, depression was not longitudinally associated with sleep quality at one-year follow-up, suggesting that this association can change depending on the moment and the specific situation of a patient. It is also possible that this discrepancy maybe related to the fact that the instrument used, the HADS, is a screening questionnaire rather than a proper diagnostic instrument for depression, with a tendency to underestimate its prevalence [38]. In fact, the presence of depressive symptom in our sample of patients with CTTH can be considered low (mean score: 8.4 points); although this mean score is similar to that one previously found in migraine [39]. It is possible that higher depressive levels would be also longitudinally associated with the quality of sleep.

The current study also supports the role of other emotional factors in sleep quality since the emotional burden of headache was also associated, but in a lower extent than depression, with sleep quality at baseline, whereas lower vitality was associated with the quality of sleep at one-year. These results suggest that other emotional aspects of the patient, and not only depression, are associated with sleep quality in CTTH. This may be related to other manifestations, such as tiredness or lack of energy, associated with poor sleep quality [40]. It seems that poor sleep quality, depression, emotional aspects, and headaches display complex relationships, and all likely influence each other in a vicious cycle.

It has been previously reported that poor sleep quality and depressive symptoms are associated with reduced pain thresholds [41]. This association is based on the fact that depression contributes to chronic pain via supraspinal pain mechanisms and emotional modulation of pain [42]. In fact, mood disorders such as depression and sleep disorders particularly sleep deprivation; can trigger hyperalgesic responses in the central nervous system [43]. The current study found that lower PPTs, i.e., higher sensitivity to pressure pain, at extra-trigeminal areas at baseline were associated with sleep quality at one year, supporting this hypothesis. Some authors have also reported that sleep disturbances and depression are independently associated with pressure pain sensitivity, supporting an independent role of each factor [44]. Our results would also agree with this proposal since depression was associated at baseline, whereas PPTs were associated longitudinally one year after, with the quality of sleep. It is possible that both variables act at different moments, or via different neural circuits, on sleep quality in individuals with CTTH.

Uncertainty over biological mechanisms withstanding in these interactions, our results have some clinical implications. Due to poor sleep quality is a common trigger of TTH, proper management of its associated factors seems to be relevant. Our study found that different associated factors are related to sleep quality at a particular time or longitudinally. In fact, all identified factors, i.e., depression, emotional aspects, and pain hypersensitivity, are modifiable with proper treatment. Therefore, the first step would be identification of these psychological, emotional, and psycho-physiological aspects in a patient with CCTH. Second, and based on current findings, therapeutic management of a patient with CTTH should include multi-modal approaches targeting depression (i.e., psychological approaches), the emotional burden of the headache (i.e., cognitive behavioral techniques) and pain sensitivity to pressure (i.e., pharmacological drugs and physical interventions targeting the excitability of the central nervous system).

Although strengths of the current study include a large sample size, a longitudinal design, the inclusion of CTTH patients accordingly the most updated diagnostic criteria, and the use of standardized instruments; we should recognize some limitations. First, we included only individuals with CTTH referred to a tertiary headache center and thus not representative of the general population; thereby, extrapolation of our results to the general population with headaches should be considered with caution. In fact, all of our patients were chronic, so these results should not be either extrapolated to patients with episodic TTH. Second, we have a drop out rate of 25% of the sample at the longitudinal follow-up period at 12 months, most of them because was not possible to contact (60%) or because they did not want to attend the appointment for personal reasons (40%). Finally, we used a specific questionnaire, e.g., the PSQI, for assessing sleep quality, but we should recognize that this questionnaire did not evaluate other sleep problems such as insomnia or obstructive sleep apnea. It is possible that these sleep disorders could have also an influence on the outcomes observed in the current study. Future studies should include specific outcomes for assessing these sleep problems, i.e., STOP-BANG sleep apnea questionnaire [45] or the Insomnia Severity Scale [46].

## Conclusions

This study found that different variables were associated with sleep quality at two different moments in individuals with CTTH in a complex interaction. Depression and the emotional burden of headache explained 27.5% of the variance in sleep quality at baseline, whereas vitality and PPTs over extra-trigeminal areas at baseline explained 30.0% of the variance of sleep quality at one year.
